# Anti‐cancer targets of formononetin and molecular mechanisms in osteosarcoma: Findings of bioinformatic and experimental assays

**DOI:** 10.1111/jcmm.14248

**Published:** 2019-03-15

**Authors:** Wei Hu, Xianpei Wu, Jiandong Tang, Guoping Zhao, Niansu Xiao, Li Zhang, Sen Li

**Affiliations:** ^1^ Spine and Osteopathy Ward Guilin Peoples’ Hospital Guilin Guangxi China; ^2^ Orthopeadics Ward Guilin Peoples’ Hospital Guilin Guangxi China

**Keywords:** bioinformatics, biomarkers, formononetin, mechanism, osteosarcoma

## Abstract

In current study, a bioinformatic‐based network pharmacology was used to identify the osteosarcoma (OGS)‐pathological targets and formononetin (FN)‐treated targets before the main core predictive biotargets were screened. In addition, all core targets were selected through a number of bioinformatic databases, followed by identification of predominant biological processes and signalling pathways of FN anti‐OGS. Further, top three core targets of FN anti‐OGS were determined as oestrogen receptor 1 (ESR1), tumour protein p53 (TP53), receptor tyrosine‐protein kinase erbB‐2 (ERBB2) respectively. In clinical biochemical data, the plasma samples of OGS showed the increased trends of alkaline phosphatase, triglyceride, blood glucose, lactate dehydrogenase, high‐sensitive C‐reactive protein and some immune cell counts when referenced to medical criteria. In clinicopathological examination, histological OGS sections resulted in increased positive cell counts of neoplastic ESR1, TP53, ERBB2. To further validate these corn proteins in experimental study in vivo, FN‐treated tumour‐bearing nude mice showed intracellular reductions of ESR1, TP53, ERBB2 positive expressions, accompanied with visibly reduced tumour weights. Collectively, our bioinformatic and experimental findings disclosed main core targets, biological processes and signalling pathways of FN anti‐OGS. Interestingly, the top core targets were representatively validated following FN treatment in vivo. Therefore, we reasoned that these predictive targets might be the potential biomarkers for screening and treating osteosarcoma.

## 1 INTRODUCTION

**Figure 1:**
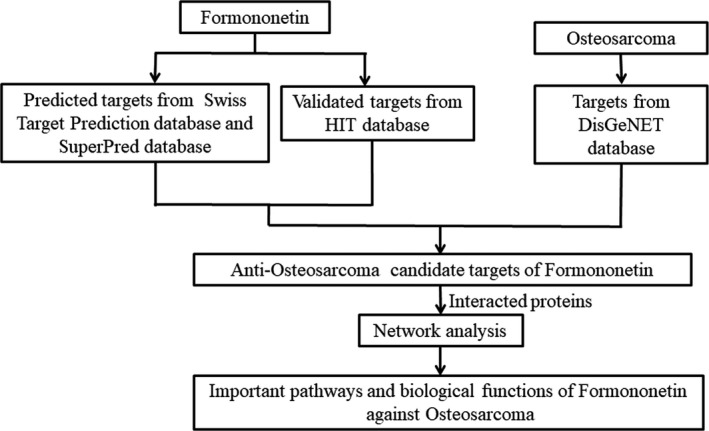
Flowchart of bioinformatic study of FN anti‐OGS using a network pharmacology strategy

Osteogenic sarcoma represents the abnormal differentiation and proliferation from bone cells, and the cells will be malignant and aggressive if untreated.^1^ Based on epidemiological data, the incidence of OGS in young individuals, accounts for 5% paediatric cancers. However, adult patients are medically diagnosed as OGS induction of genetic mutation or environmental factor.^2^ As a clinical precaution, early predictive inspection of OGS may be achieved through biochemical, imaging, pathological tests.^3^ In clinical practice, routine anti‐cancer and targeted drugs are prescribed commonly, followed by unwanted adverse effects and chemoresistance.^4^ Thus, our task is to pursue a safe and effective medication for combating OGS. Historically, traditional Chinese medicine (TCM) is demonstrated with therapeutic efficacy against cancers, as revealed by less side effects.^5^ TCM in the treatment of bone cancer pain is used in China before the TCM‐rich component is isolated for anti‐cancer therapy.^6^ Formononetin (FN), a bioactive chemical from *Astragalus root*, is found to play many benefits, such as anti‐neuropathy, anti‐hyperlipaemia and anti‐cardiopathy effects.^7^ In another pharmacological activity, FN can prevent cancers through regulating oestrogen‐dependent signalling pathway, including breast, prostate and colon cancers.^8^ Additionally, our previous data showed that FN promotes cell apoptosis of human bone cancer in vitro and in vivo through the molecular mechanism of regulating intracellular Bcl‐2, Bax and MiR‐375 expressions.^9^ However, detailed molecular targets of FN anti‐OGS have not yet totally identified. Recently, network pharmacology, a bioinformatic method, can be used to elucidate the predictive targets and pharmacological mechanisms of TCM‐isolated component.^10^ Methodologically, the data of network pharmacology can help pharmacologist to disclose the therapeutic biomarkers and mechanisms of FN anti‐OGS. And currently no report has explored this topic using network pharmacology for studying FN anti‐OGS. As a result, our present study was designed to use the method of network pharmacology to construct a network of FN anti‐OGS in targets‐OGS‐pathways interactions for predicting the therapeutic targets and pharmacological mechanisms respectively. Further, these predictive targets would be validated by human OGS samples, and FN‐treated tumour‐bearing nuke mice model. First of all, the flowchart of investigative designs in this study was revealed in Figure 1.

## 2 MATERIALS AND METHODS

### 2.1 Collection of pharmacological targets in FN anti‐OGS

The herbal ingredients targets (HIT) database was employed to collect the associated targets of FN. In addition, treated targets of FN were predicted via two different databases of chemical association networks (STITCH) and Swiss TargetPrediction. Further, a database of gene‐disease associations (DisGeNET) was utilized to screen the OGS‐diseased genes, and the optimal genes were identified through the designed parameters and scoring index. Subsequently, the optimal targets of FN were pooled with the disease genes of OGS before producing pharmacological targets of FN anti‐OGS.

### 2.2 Confirmation of protein‐protein interaction (PPI) network and customization of core targets

The pick‐up targets were re‐assay using a database of functional protein association networks (STRING) to further produce a PPI network of FN anti‐OGS. Following computerized setting, correlative proteins with a confidence score >0.9 were identified via a Cytoscape software for eliminating duplicates, mean degree of freedom and maximum degree of freedom. And a target‐associated protein network of FN anti‐OGS was produced. After collecting a target‐related protein network, core targets of FN anti‐OGS were produced through grading values in software parameters.

### 2.3 Data of core targets‐associated biological functions and pathway

Following by Funrich software, the core targets of FN anti‐OGS were identified the correlative biological processes and signalling pathways via enrichment analyses. Among these data, top 20 biological processes and signalling pathways were isolated according to the degree of importance for further discussion.

### 2.4 Human study of clinical cases of OGS

In hospitalization cases, five adult patients were medically identified as OGS using biochemical test and pathological inspection during 2018. The biochemical data of OGS patients were harvested for analysis. In addition, histopathological sections of OGS were used for immunofluorescence staining. Further, some OGS and OGS‐free samples were surgically isolated and stored at liquid nitrogen before conducting western blot experiment. And the Hospital Ethics Committee has approved this human study. This human study was implemented according to the required principles of the Declaration of Helsinki.^11^

### 2.5 Pharmacological study of FN against tumour‐bearing mice

Commercially available BALB/c‐nu nude mice, aged 6 weeks, were purchased from Hunan Slack Jingda Experimental Animal Co., Ltd (Changsha, China). After being acclimatized for 5 days, the mice were hypodermically injected with U2‐OS cells (a human OGS cell line, ATCC, USA) at a cell density of 1 × 10^7^. After being producing solid tumour, the tumour‐bearing mice were treated with different doses of FN for 14 days. At the end of the experiments, the tumour was removed and weighed. Some of the tumour samples were fixed with 4% paraformaldehyde for immunostaining.^12^

### 2.6 Immunofluorescence staining procedures

As described previously,^13^, ^14^ both human OGS and mice tumour samples were cut as 5‐μm sections before dewaxing. The sections were further blocked with 5% bovine serum albumin (BSA) solution for 1 hour, and then were incubated with primary antibodies of ESR1, TP53, ERBB2 (1:50, Bioss, Beijing, China) at 4°C overnight. After being washed with PBS/0.5% tween20, the sections were incubated with Alexa Fluor 488‐conjugated secondary antibody (Beyotime, Nanjing, China). The nuclei were developed with 4,6‐diamidino‐2‐phenylindole (DAPI) prior to imaging using an inverted fluorescence microscope (Olympus, Japan). The average fluorescence‐positive cells (green) in each section were calculated through at least three different views under 200 × magnification.

## 3 RESULTS

### 3.1 The characteristics of biological targets

**Figure 2:**
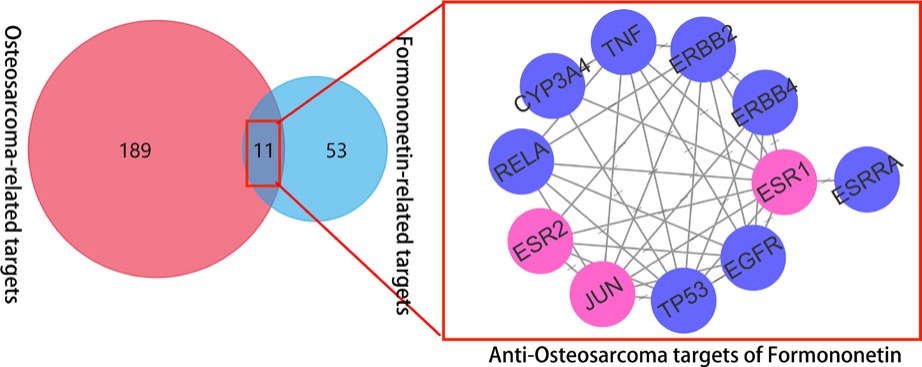
The detailed network of OGS‐diseased targets and FN‐associated targets

**Figure 3:**
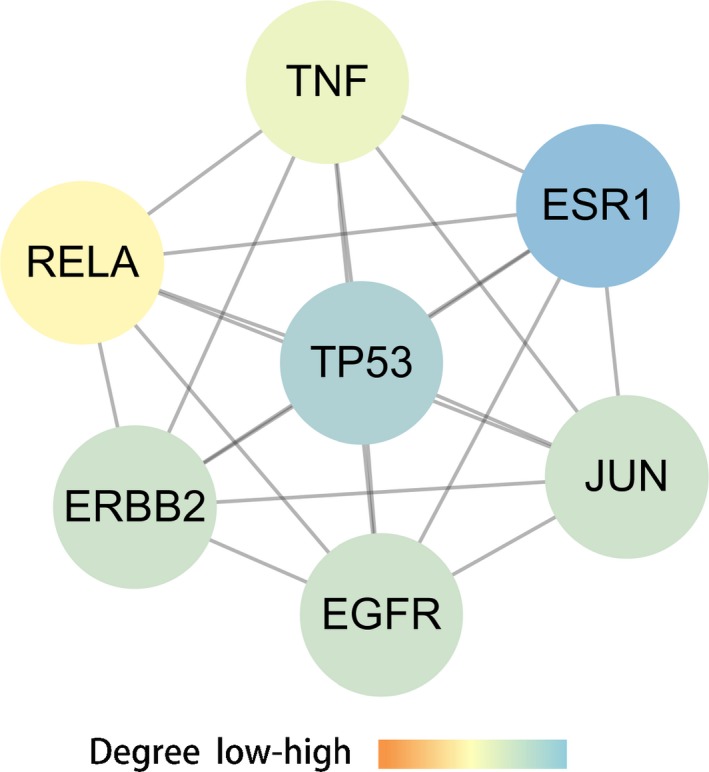
Network of correlative core targets. And the top targets were showed as ESR1, TP53, ERBB2, Jun, EGFR, TNF, RELA respectively

As a result, total 200 genes from DisGeNET database were harvested widely. Meanwhile, other 189 diseased genes of OGS were pick‐up as potential targets for re‐analysis. In addition, 53 FN‐associated genes from the HIT database were identified. As a result, 12 therapeutic targets of FN anti‐OGS were finally identified through pooled data of diseased and pharmacological targets (Figure 2). In PPI data and network topology assay, the results showed that the main core targets were produced as ESR1, TP53, ERBB2, Jun, EGFR, TNF, RELA respectively (Figure. 3).

### 3.2 Main biological processes and signalling pathway of FN anti‐OGS

**Figure 4:**
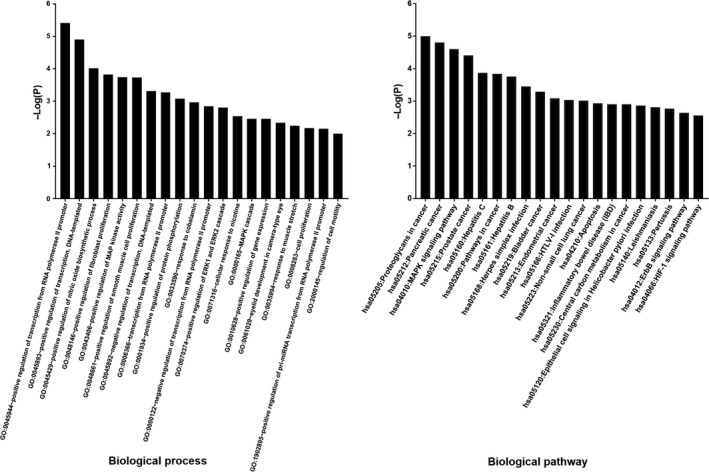
Main biological processes and molecular pathways of FN anti‐OGS. Both top 20 biological processes and biological signalling pathways of FN anti‐OGS were characterized and identified respectively

As shown in Figure 4, the biological processes of FN anti‐OGS were mainly related to the regulation of cell motility, cell proliferation, positive regulation of gene expression, MAPK cascade, positive regulation of ERK1 and ERK2 cascade and positive regulation of MAP kinase activity. In addition, other top signalling pathways were confirmed respectively. And the dominating biological signalling pathways were basically associated with the MAPK signalling pathway, some cancer pathways, apoptosis, central carbon metabolism in cancer, ErbB signalling pathway, HIF‐1 signalling pathway.

### 3.3 Clinicopathological characteristics of OGS patients

**Figure 5:**
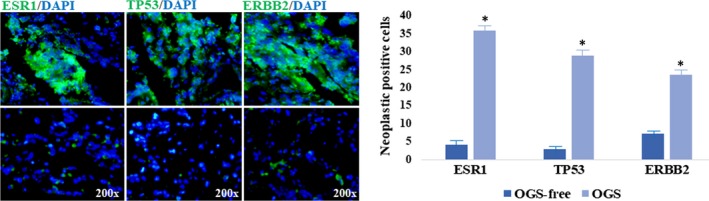
Preliminary findings of patients with OGS. Data from quantitative immunofluorescent assay showed markedly increased neoplastic positive cells of ESR1, TP53, ERBB2 in OGS sections in comparison with those in OGS‐free sections Note: Compared to control, *P <* *0.05

In baseline data, the adult patients with OGS exhibited that the average age was (30.60 ± 17.5) years and the sexual proportion was Male:Female (4:1). As shown in Table 1, the increased trends of alkaline phosphatase, triglyceride, blood glucose, lactate dehydrogenase, high‐sensitive C‐reactive protein, some immune cell counts in blood samples of OGS were observed. However, inapparent changes of trace element, hepatonephric functional enzymes and cancer antigen contents were showed. To validate the top three targets on human OGS samples, quantitative immunofluorescent analysis was conducted. Compared to OGS‐free controls, neoplastic positive cells of ESR1, TP53, ERBB2 in OGS sections were increased respectively. And the positive cells in OGS cases were greater than those in OGS‐free sections (*P *<* *0.05) (Figure 5).

**Table 1:** Preliminary clinical data in patients with osteosarcoma

Clinical parameters	OGS patients	Medical references
Gender (M/F)	4/1	–
Age (year)	30.60 ± 17.48	–
WBC (109/L)	9.80 ± 5.50	4‐10
NEUT (109/L)	75.16 ± 13.02	50‐70
LY (109/L)	17.74 ± 11.02	20‐40
MONO (109/L)	5.32 ± 3.62	3‐10
EO (109/L)	0.92 ± 0.78	0.5‐5
BASO (109/L)	0.24 ± 0.33	0‐1
RBC (1012/L)	4.84 ± 0.41	4‐5.5
HGB (g/L)	127.40 ± 16.21	120‐160
PLT (109/L)	302.40 ± 91.59	100‐300
Ca (mmol/l)	2.44 ± 0.17	2.1‐2.6
Mg (mmol/l)	0.97 ± 0.05	0.67‐1.04
*P* (mmol/l)	1.05 ± 0.15	0.96‐1.62
GLU (mmol/L)	6.32 ± 0.74	3.9‐6.1
AMY (μmol/L)	121.80 ± 28.41	0‐220
Urea (mmol/L)	4.75 ± 1.55	1.7‐8.3
Cr (μmol/L)	68.20 ± 24.14	53‐106
UA (μmol/L)	307.00 ± 63.17	150‐420
CHO (mmol/L)	4.45 ± 0.63	3.12‐6.24
TG (mmol/L)	1.49 ± 0.88	<1.71
HDL‐C (mmol/L)	1.31 ± 0.19	0.91‐1.56
LDL‐C (mmol/L)	2.39 ± 0.43	<3.5
hsCRP (mg/L)	14.20 ± 13.12	<3.0
LDH‐L (U/L)	244.00 ± 123.35	115‐220
ALT (U/L)	33.20 ± 21.87	0‐40
AST (U/L)	27.60 ± 5.61	<40
ALP (U/L)	142.40 ± 65.37	40‐150
AFP (ng/mL)	4.84 ± 3.73	0‐25
CEA (ng/mL)	1.66 ± 1.09	0‐6.5
CA125 (U/mL)	12.51 ± 2.52	0‐35
CA153 (U/mL)	13.25 ± 6.19	0‐25
CA199 (U/mL)	8.15 ± 3.93	0‐37

Note

AFP, alpha‐fetoprotein; ALP, alkaline phosphatase; ALT, alanine transaminase; AMY, amylase; AST, aspartate transaminase; BASO, basophil; CA125, 153,199, cancer antigen 125, 153, 199; CEA, carcinoembryonic antigen; CHO, cholesterol; EO, eosinophil; F, female; GLU, glucose; HDL‐C, high‐density lipoprotein cholesterol; HGB, haemoglobin; hsCRP, hypersensitive C‐reactive protein; LDH, lactate dehydrogenase; LDL‐C, low‐density lipoprotein cholesterol; LY, lymphocyte; M, male; MONO, monocyte; NEUT, neutrophil; PLT, platelet parameter; RBC, red blood cell; TG, triglyceride; UA, uric acid; WBC, white blood cell.

### 3.4 Anti‐OGS effects of FN on tumour‐bearing nude mice

**Figure 6:**
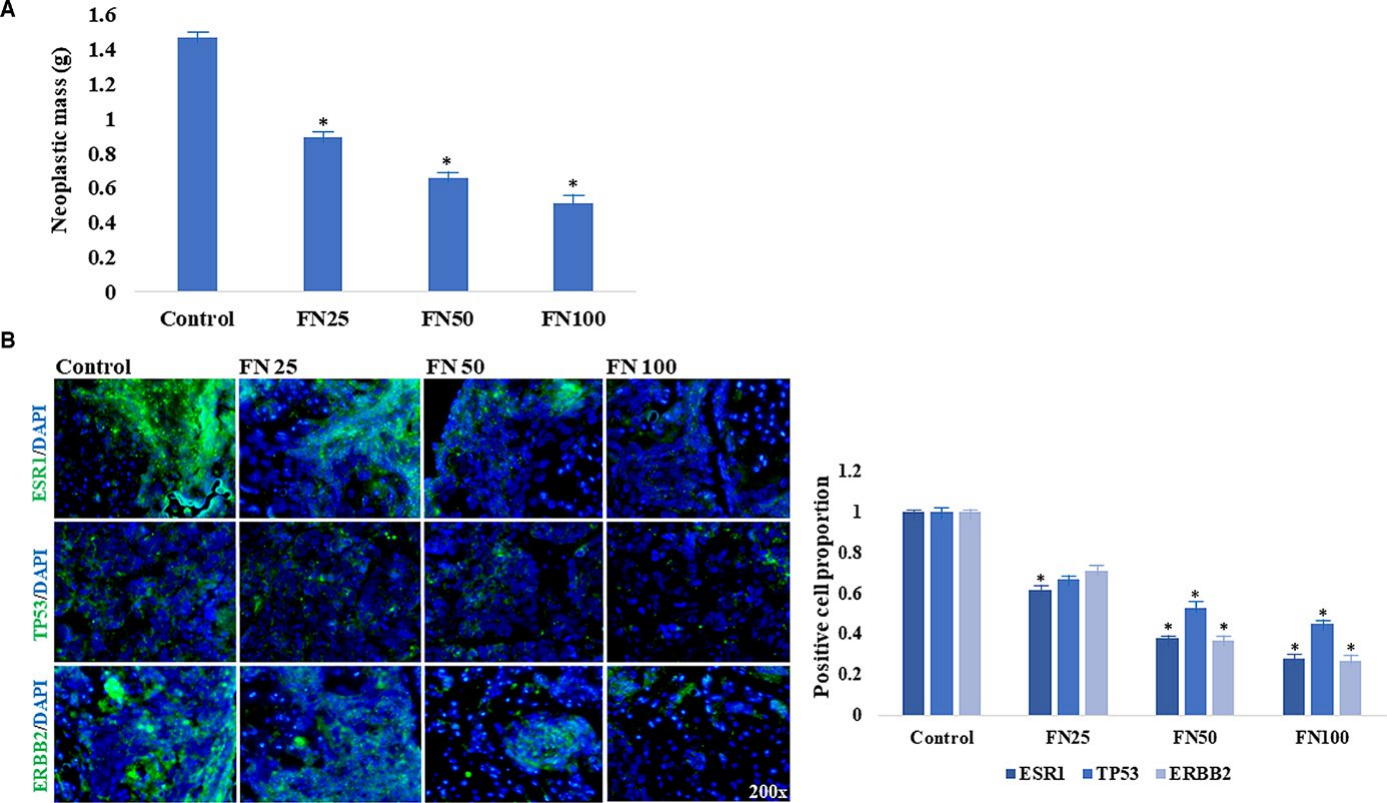
Anti‐cancer effects of FN against OGS in vivo. As a result, FN‐treated tumour‐bearing nude mice showed lowered tumour weights in a dose‐dependent manner (A). Further, immunostaining analysis indicated that intracellular down‐regulations of ESR1, TP53, ERBB2 expressions in FN‐treated mice were observed in a dose‐dependent manner (B). Note: Compared to control, *P <* *0.05. FN25, 50, 100 =  formononetin 25, 50, 100 mg/kg per day for 2 wk

To characterize the pharmacological activities of FN on OGS, the transplantable tumour mice were used to validate the predictive targets. Beneficially, FN‐treated mice resulted in reduced tumour mass in a dose‐dependent manner (*P *<* *0.05) (Figure 6A). In addition, immunofluorescence staining assay showed that dose‐dependent reductions of intracellular ESR1, TP53, ERBB2 positive cells were observed following FN treatments respectively. And these positive cell counts in FN groups were lower than those in controls (*P *<* *0.05) (Figure 6B).

## 4 DISCUSSION

A primary osteosarcoma is a neoplastic growth in bone tissue, followed by malignant proliferation and metastasis.^15^ Clinically, the effectiveness and safeness of chemotherapy for OGS are limited. Therefore, developing alternative medication against OGS is urgent and required. Formononetin , a potent phytoestrogen, is demonstrated with promising pharmacological effects, including anti‐neoplastic action.^16^ Our previous findings suggest that FN exerts effective activities for inhibiting OGS in vitro and in vivo. However, the optimal biological targets and mechanisms remain unknown. Thus, our present study aimed to prioritize bioinformatic‐based network pharmacology to disclose the therapeutic targets and bio‐mechanisms of FN anti‐OGS. Followed by database‐assayed data, the biological processes of FN anti‐OGS were principally linked to the regulation of cell motility, cell proliferation, positive regulation of gene expression, MAPK cascade, positive regulation of ERK1 and ERK2 cascade, positive regulation of MAP kinase activity. These functional processes were associated with the development of OGS, indicating that FN might play the anti‐OGS benefits by regulating the main biological processes proposed. Further, the major biological signalling pathways were basically associated with the MAPK signalling pathway, some cancer pathways, apoptosis, central carbon metabolism in cancer, ErbB signalling pathway, HIF‐1 signalling pathway. As a result, we finally identified three core targets of FN anti‐OGS for human and experimental validation. ESR1 (oestrogen receptor 1) is a nuclear receptor for hormone binding, DNA binding and activation of transcription. And ESR1 modulates breast cancer program physiological events, such as growth, apoptosis, migration or invasion.^17^ TP53 (tumour protein p53) functions as a tumour suppressor. TP53 has many mechanisms of anti‐cancer function and plays a role in apoptosis, genomic stability and inhibition of angiogenesis. However, TP53 mutation has been associated with tumourigenesis and carcinomatous metastasis.^18^, ^19^ ERBB2 (receptor tyrosine‐protein kinase erbB‐2) is a member of the epidermal growth factor receptor that promotes cell proliferation and opposes apoptosis, and therefore must be tightly regulated to prevent uncontrolled cell growth.^20^ In human study, elevated positive proteins of neoplastic ESR1, TP53, ERBB2 were observed in OGS samples, followed by abnormal cancer antigen contents in blood samples. Based on the literature and human data, we further conducted the pharmacological experiments of FN OGS using a tumour‐bearing nude mouse model. As a result, FN‐treated mice showed reduced intracellular ESR1, TP53, ERBB2 expressions in a dose‐dependent manner, characterized with lowered tumour weights. Together, these current findings demonstrated the FN‐lowering ESR1 expression for suppression of cell proliferation, FN‐reducing TP53 expression for inhibition of gene mutation and tumour growth, and FN‐decreasing ERBB2 expression for blocking neoplastic development and metastasis. And these anti‐OGS benefits were consistent with the main biological processes and signalling pathways proposed. Beneficially, these predictive biotargets might be likely the potential biomarkers for screening and treating OGS.

## 5 CONCLUSION

Taken together, these bioinformatic findings from network pharmacology discolse the main biotargets and molecular mechanisms of FN anti‐OGS. In addition, further clinicopathological and experimental data are validated to characterize the top core targets. Therefore, the network pharmacology may be used for development of TCM‐isolated component.

## CONFLICT OF INTEREST

The authors declare no conflict of interest and are responsible for the contents of this study.
